# Stochastic thermodynamics for biological functions

**DOI:** 10.1002/qub2.75

**Published:** 2024-12-16

**Authors:** Yuansheng Cao, Shiling Liang

**Affiliations:** ^1^ Department of Physics Tsinghua University Beijing China; ^2^ Institute of Physics School of Basic Sciences École Polytechnique Fédérale de Lausanne (EPFL) Lausanne Switzerland

**Keywords:** biological functions, physical constraints, stochastic thermodynamics

## Abstract

Living systems operate within physical constraints imposed by nonequilibrium thermodynamics. This review explores recent advancements in applying these principles to understand the fundamental limits of biological functions. We introduce the framework of stochastic thermodynamics and its recent developments, followed by its application to various biological systems. We emphasize the interconnectedness of kinetics and energetics within this framework, focusing on how network topology, kinetics, and energetics influence functions in thermodynamically consistent models. We discuss examples in the areas of molecular machine, error correction, biological sensing, and collective behaviors. This review aims to bridge physics and biology by fostering a quantitative understanding of biological functions.

## 1 INTRODUCTION

Physics and biology, while sharing a common goal of understanding the natural world, employ distinct paradigms and terminologies to achieve this objective. Physics strives to identify fundamental, universal laws governing the behavior of matter and energy, often expressed using concepts such as energy, entropy, forces, and rates. In contrast, biology delves into the intricate complexities of life, focusing on the structures, functions, and information flow within living systems, often utilizing terms such as signals, codes, transcription, and translation.

Modern biological physics, or the physics of living systems, bridges the disciplinary gap by applying the principles and methodologies of physics to elucidate biological phenomena. A central focus of this interdisciplinary field is the investigation of biological functions. From the molecular machinery within a cell to the biomechanics of organismal movement, biological physics seeks to unravel the underlying physical principles governing these processes.

The functionality of living systems is constrained by the fundamental laws of physics, chemistry, and biology. Within signaling networks, for example, receptor specificity ensures that they only respond to specific ligands. This biochemical specificity arises from the molecular structures shaped by evolution. However, as will be detailed later, the fundamental limitations governing many biological functions stem not only from biological and chemical factors but also from the basic physical principles governing cellular processes and their environment.

Among the relevant subfields of physics, thermodynamics provides some of the most universal constraints on biological systems. For instance, the conservation of energy (the first law of thermodynamics) and material limits the yield of cellular metabolism [1, 2]. The second law of thermodynamics, which states that isolated systems tend toward maximum disorder (and therefore incompatible with life), has long challenged physicists seeking to understand how living systems maintain their ordered state. Erwin Schrödinger, in his influential book *What is Life?*, asserted that living cells must absorb “negative entropy” or “free energy” as it is termed in standard thermodynamics, to counterbalance the entropy produced within biological processes and maintain their low‐entropy (highly ordered) states. Thermodynamically, a key distinction between a cell and a box of gas is that a cell represents an open system, continuously exchanging energy with its surroundings to remain in a state of dynamic order.

Traditional equilibrium thermodynamics offers a well‐suited framework for describing a box of gas molecules. However, due to their continuous exchange of free energy with the environment, cells are inherently nonequilibrium systems. Notably, a central theme of this review is that the cost of free energy imposes fundamental limits on the performance of biological functions. Furthermore, cellular systems often operate at the microscopic or mesoscopic scale, where the number of participating molecules, typically ranging from 10 to 10^5^, falls well below the thermodynamic limit of 10^23^ particles. This characteristic classifies a typical cell as a microscopic or mesoscopic nonequilibrium system. Consequently, the performance of biological functions is also inherently limited by the inevitable fluctuations arising from the small number of molecules involved.

Recent advances in stochastic thermodynamics provide a powerful tool to address the two features listed above. Stochastic thermodynamics investigates the behavior of mesoscopic systems governed by thermal fluctuations, both in and out of equilibrium, establishing a fundamental relationship between thermodynamic principles and stochastic kinetics. This field traces its roots to Einstein’s seminal work on Brownian motion, in which he established the fluctuation–dissipation theorem (FDT), revealing a profound connection between fluctuation and dissipation at thermodynamic equilibrium. For systems out of equilibrium (such as a Brownian particle coupled to multiple thermal reservoirs or subjected to time‐dependent environmental changes), a broader thermodynamic framework is necessary, particularly for understanding inherently nonequilibrium biological processes at the subcellular level. From the latter half of the 20th century, significant efforts were made to connect the irreversible dynamics of stochastic processes with entropy production [3, 4, 5], leading to a comprehensive framework with precise definitions of thermodynamic quantities like work and heat at the mesoscopic level [6]. Building upon this framework, researchers have applied advanced mathematical and physical tools to investigate fundamental thermodynamic constraints, resulting in key findings such as the fluctuation theorem which quantifies the irreversibility of mesoscopic systems [7, 8, 9], the thermodynamic uncertainty relation (TUR) which identifies the thermodynamic cost of suppressing current fluctuations [10], and nonequilibrium response bounds demonstrating universal constraints on the sensitivity of nonequilibrium response [11]. These advances in stochastic thermodynamics illuminate the thermodynamic principles governing mesoscopic stochastic systems and set physical constraints on mesoscopic biological processes.

This review delves into recent advancements in understanding the physical limitations, particularly from a nonequilibrium thermodynamics perspective, on the performance of various biological functions. We begin by introducing the fundamental framework and recent developments in stochastic thermodynamics. Subsequently, we showcase advancements in applying these principles to diverse biological systems. Recognizing the deep connection between kinetics and energetics in stochastic thermodynamics (e.g., local detailed balance [LDB]), the review specifically focuses on studies that elucidate how kinetics, energetics, and network topology determine biological function within thermodynamically consistent models. Three key questions will be addressed and discussed: (1) how to define a specific biological function within the context of biological networks and thermodynamics, (2) how stochastic thermodynamics, particularly free energy dissipation (or entropy production), imposes constraints on biological functionality, and (3) how to approach these limitations and optimize performance. These key aspects will be examined with specific examples in various areas, including the precision of molecular machines, error correction mechanisms, biological sensing, and the emergence of collective behaviors. This review aims to bridge the gap between physics and biology, fostering a quantitative understanding of biological functions.

## 2 INTRODUCTION TO STOCHASTIC THERMODYNAMICS

### 2.1 Thermodynamics of stochastic processes

Many biological processes, such as molecular motor motion, transcription, and protein folding, can be modeled as Markovian dynamics where thermal noise triggers transitions between mesoscopic states [12, 13, 14, 15]. Stochastic thermodynamics offers a comprehensive framework for describing the thermodynamics of stochastic processes by establishing constraints on kinetics. Consequently, it enables precise definitions of thermodynamic quantities, such as entropy production, at both trajectory and ensemble levels [16, 17, 18]. This framework further allows for the derivation of thermodynamic constraints on various physical observables, illustrating how physical laws limit biological functions. Here, we will introduce the basic framework of stochastic thermodynamics and highlight several recent advances in this field.

#### 2.1.1 Master equation

A discrete‐state stochastic process is governed by a master equation that describes the time evolution of the occupation probabilities of states:

(1) $$\frac{\text{d}}{\text{d}t}p(t)=Wp(t),$$

where **p**(*t*) = [*p*_1_(*t*), *p*_2_(*t*), …, *p*_*n*_(*t*)] represents the vector of probability distribution across all states in the system at time *t*. Here, *W* is the transition rate matrix, with its element *W*_*ij*_ indicating the transition rate from state *j* to state *i*. The time evolution of the probability for state *i* can be derived from the equation as follows:

(2) $$\frac{\text{d}}{\text{d}t}p_{i}=\sum_{j\neq i}\left(W_{ij}p_{j}-W_{ji}p_{i}\right),$$

where *J*_*ij*_ = *W*_*ij*_ *p*_*j*_ represents the probability current from state *j* to state *i*, contributed by all transition events along the edge *e*_*ij*_ from *j* to *i*. The net current from state *j* to state *i* is $J_{ij}=J_{ij}-J_{ji}$. In the long time limit, the system reaches a stationary state, denoted by **p** ^st^, in which all net currents are balanced, such that $\sum_{j\neq i}J_{ij}^{\text{st}}=0$ for any *i* or in matrix product form *W* **p** ^st^ = 0. If all net currents are zero, that is, $J_{ij}^{\text{st}}=0$ for all pairs *i*, *j*, the steady state is an equilibrium state.

**FIGURE 1:**
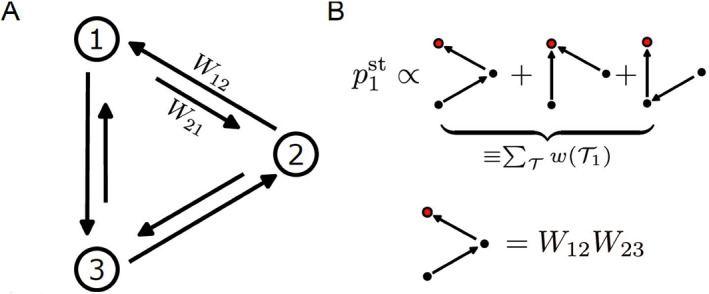
Graph‐theoretic solution for Markov chain. (A) A three‐state Markov chain. (B) The graph‐theoretic solution of stationary state probability.

The steady‐state probability distribution can be derived using a graph‐theoretic approach, expressed as follows:

(3) $$p_{i}^{\text{st}}=\frac{\sum_{T}w\left(T_{i}\right)}{\sum_{j}\sum_{T}w\left(T_{j}\right)},$$

where $T_{i}$ is a directed spanning tree rooted at state *i*, and $w\left(T_{i}\right)$ is the weight of the directed tree $T_{i}$, calculated as the product of the transition rates along the edges of the tree. Figure 1 illustrates the spanning tree representation of the steady‐state probability distribution for a 3‐state system.

This graph‐theoretic solution has been discovered several times throughout history [19, 20, 21]. It circumvents the direct computation of matrix inversions and facilitates theoretical analysis based on the graph‐theoretic properties of the network. This analysis serves as a foundational element for exploring the thermodynamics of stochastic processes. Such explorations have led to numerous results, including cycle‐decomposition of entropy production [20], bounds on nonequilibrium responses [11, 22], constraints on symmetry breaking in biochemical system [23], and the development of the spanning‐tree representation for first passage times statistics in Markov chains [21, 24].

#### 2.1.2 Local detailed balance

For small systems, the principle of microscopic reversibility mandates that for any transition, an associated backward transition must exist. In a system governed by the master equation, thermodynamics is introduced on every pair of transition rates through the LDB condition [18, 25],

(4) $$\frac{W_{ij}}{W_{ji}}={\text{e}}^{\Delta S_{ij}^{\text{env}}/k_{\text{B}}},$$

where *k*_B_ is the Boltzmann constant and $\Delta S_{ij}^{\text{env}}$ represents the entropy production into the environment for the transition *j* → *i*. The entropy production into the environment is determined by the energy exchange between the system and its environment, incorporating two contributions: the energy difference between the two states, *ϵ*_*j*_ − *ϵ*_*i*_, and the driving force *F*_*ij*_,

(5) $$\Delta S_{ij}^{\text{env}}=\left(\epsilon_{j}-\epsilon_{i}\right)/T+F_{ij},$$

where *T* is the temperature of the environment. The entropy production quantifies the irreversibility of a transition—if the entropy production is positive (i.e. the transition from state *j* to state *i* increases the entropy of the environment), then *W*_*ij*_ > *W*_*ji*_, indicating a preference for the forward transition over the backward one. The local thermodynamic definition of transition rates allows us to assess whether a system is in or out of equilibrium on a global level. One can calculate the nonequilibrium driving force along a cycle *c* = [*m*_0_, *m*_1_, *m*_2_, …, *m*_*n*_, *m*_0_] in the network,

(6) $$F_{c}=\ln\left(\frac{W_{m_{0},m_{1}}W_{m_{1},m_{2}}\text{…}W_{m_{n},m_{0}}}{W_{m_{1},m_{0}}W_{m_{2},m_{1}}\text{…}W_{m_{0},m_{n}}}\right),$$

referred to as cycle affinity, or cyclic driving force. When *F*_*c*_ ≠ 0, the time‐reversal symmetry is broken, indicating the system is out of equilibrium—as traversing a cycle results in nonzero entropy production. Conversely, if *F*_*c*_ = 0 for all cycles in the network, known as Kolmogorov’s criterion [26], the system is an equilibrium system and preserves time‐reversal symmetry. The equilibrium nature of a system allows the construction of an energy landscape in which the entropy production of a transition is solely determined by the energy difference between the initial and final states, that is, $\Delta S_{ij}^{\text{env}}=\left(\epsilon_{j}-\epsilon_{i}\right)/T$. Consequently, the LDB condition reduces to the detailed balance condition

(7) $$\frac{p_{i}^{\text{eq}}}{p_{j}^{\text{eq}}}=\frac{W_{ij}}{W_{ji}}={\text{e}}^{-\beta \left(\epsilon_{i}-\epsilon_{j}\right)},$$

for any pair of states, where *β* = 1/(*k*_B_ *T*) is the inverse temperature. Detailed balance ensures the system can relax to an equilibrium Boltzmann distribution,

(8) $$p_{i}^{\text{eq}}=\frac{{\text{e}}^{-\beta \epsilon_{i}}}{\sum_{j}{\text{e}}^{-\beta \epsilon_{j}}},$$

where the summation in the denominator is over all states. With detailed balance, the graph‐theoretical solution, Equation (3), can be reduced to the Boltzmann distribution.

#### 2.1.3 Entropy production rate

The LDB condition links the kinetics of a Markov process with its thermodynamic properties. Beyond the entropy production of individual transitions, it is possible to define the entropy production rate (EPR) at the ensemble level, which quantifies the average rate of entropy production across the entire system. Denoting the EPR as $\dot{\Sigma }$, it can be expressed as follows:

(9) $$\begin{array}{cc} {\dot{\Sigma }}_{\text{tot}} & =k_{\text{B}}\sum_{i,j>i}J_{ij}\;\ln\frac{J_{ij}}{J_{ji}}\\ & =\underset{\text{environment}\;\text{EPR,}\;{\dot{\Sigma }}_{\text{env}}}{\underbrace{k_{\text{B}}\sum_{i,j>i}J_{ij}\;\ln\frac{W_{ij}}{W_{ji}}}}+\underset{\text{system}\;\text{EPR,}\;{\dot{\Sigma }}_{\text{sys}}}{\underbrace{k_{\text{B}}\sum_{i,j>i}J_{ij}\;\ln\frac{p_{j}}{p_{i}}}}. \end{array}$$

The total EPR, ${\dot{\Sigma }}_{\text{tot}}$, can be decomposed into the environment EPR, ${\dot{\Sigma }}_{\text{env}}$, and the system EPR, ${\dot{\Sigma }}_{\text{sys}}$. Notably, the system EPR equals the time derivative of the system’s Shannon entropy, ${\dot{\Sigma }}_{\text{sys}}=\frac{\text{d}}{\text{d}t}\left(-k_{\text{B}}\sum_{i}p_{i}\;\ln p_{i}\right)$. At a nonequilibrium stationary state, the probability distribution remains unchanged over time, resulting in zero system EPR. The sign of $J_{ij}=J_{ij}-J_{ji}$ always aligns with that of ln(*J*_*ij*_ /*J*_*ji*_), ensuring the non‐negative total EPR. This property is consistent with the second law of thermodynamics, which states that the total entropy of an isolated system (here the system and environment together constitute an isolated system) is a non‐decreasing function.

### 2.2 Stochastic trajectories and fluctuation theorems

**FIGURE 2:**
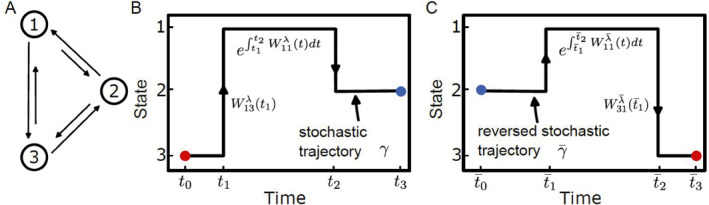
Stochastic trajectories for a Markov chain. (A) A three‐state Markov chain. (B) A stochastic trajectory on the three‐state network. The probability of a trajectory contains the contribution from transition events and the survival probability among the states along the trajectory. (C) The time‐reversed trajectory.

The master equation provides a deterministic description of the time evolution of probability distributions in a Markov chain, obtained by averaging over the many possible stochastic trajectories which are generated by the transition rate matrix *W*. On the other hand, the Markov chain itself models the random transitions between states, capturing the inherent fluctuations at the level of individual trajectories. As depicted in Figure 2B, a stochastic trajectory (and the time‐reversed trajectory in Figure 2C) within a three‐state network provides a visual representation of these concepts. Understanding the thermodynamic properties of stochastic trajectories and extracting information from them are central problems in stochastic thermodynamics. To address these challenges, we first introduce the probability of a stochastic trajectory for a general discrete‐state stochastic process. In the most general case, a system can be subjected to external control, *λ*_*t*_, leading to a time‐dependent transition matrix, *W* ^*λ*^(*t*). A stochastic trajectory is a sequence of states [*γ*_0_, *γ*_1_, …, *γ*_*n*_] along with the corresponding times of transition events [*t*_0_, *t*_1_, *t*_2_, …, *t*_*n*_, *t*_*n*+1_], where *t*_0_ = 0 and *t*_*n*+1_ = *τ* denote the initial and final times of the trajectory, respectively, and each *t*_*i*_ represents the time for the transition *γ*_*i*−1_ → *γ*_*i*_. The probability of a trajectory can be written as follows:

(10) $$P_{\gamma }=p\left(\gamma_{0},0\right)\prod_{i=1}^{n}W_{\gamma_{i}\gamma_{i-1}}^{\lambda }\left(t_{i}\right)\prod_{i=0}^{n}e^{\int_{t_{i}}^{t_{i+1}}W_{\gamma_{i}\gamma_{i}}^{\lambda }(t)\text{d}t},$$

where *p*(*γ*_0_, 0) is the probability at state *γ*_0_ with the initial distribution, $W_{\gamma_{i}\gamma_{i-1}}^{\lambda }\left(t_{i}\right)$ is the probability of transition *γ*_*i*−1_ → *γ*_*i*_ at time *t*_*i*_, and $e^{\int_{t_{i}}^{t_{i+1}}W_{\gamma_{i}\gamma_{i}}(\lambda (t))\text{d}t}$ is the survival probability on state *γ*_*i*_ between the in and out transitions. Denoting the probability of the reversed trajectory $\bar{\gamma }$ under time‐reversed protocol $\bar{\lambda }\left(t\right)=\lambda \left(t_{n+1}-t\right)$ as ${\bar{P}}_{\bar{\gamma }}$, one can find that the ratio of these two probabilities is determined by the total entropy production along the trajectory [27].

(11) $$\frac{P_{\gamma }}{{\bar{P}}_{\bar{\gamma }}}={\text{e}}^{\Delta S_{\gamma }^{\text{tot}}/k_{\text{B}}},$$

where,

(12) $$\Delta S_{\gamma }^{\text{tot}}=k_{\text{B}}\;\ln\frac{p\left(\gamma_{0},0\right)}{p\left(\gamma_{n},\tau \right)}+k_{\text{B}}\;\ln\frac{\prod_{i=1}^{n}W_{\gamma_{i}\gamma_{i-1}}^{\lambda }\left(t_{i}\right)}{\prod_{i=1}^{n}W_{\gamma_{i-1}\gamma_{i}}^{\lambda }\left(t_{i}\right)}.$$

Equation (11) quantifies the irreversibility on trajectory level, and can also be understood as a direct consequence of LDB condition [25]. By integrating over all trajectories with equal entropy production, we can find the detailed fluctuation theorem [28].

(13) $$\frac{P\left(\Delta S^{\text{tot}}\right)}{\bar{P}\left(-\Delta S^{\text{tot}}\right)}={\text{e}}^{\frac{\Delta S^{\text{tot}}}{k_{\text{B}}}},$$

which states that the probability of observation of entropy production of an amount Δ*S* ^tot^ is ${\text{e}}^{\Delta S^{\text{tot}}/k_{\text{b}}}$ more likely than observing the same amount of negative entropy production under a time‐reversal control protocol. This is one of the most fundamental relations in stochastic thermodynamics. By averaging ${\text{e}}^{-\Delta S_{\gamma }^{\text{tot}}/k_{\text{B}}}$ over all possible trajectories, one can obtain the integrated fluctuation theorem,

(14) $${\langle {\text{e}}^{-\frac{\Delta S_{\gamma }^{\text{tot}}}{k_{\text{B}}}}\rangle }_{\gamma }=\int_{\gamma }P_{\gamma }{\text{e}}^{-\frac{\Delta S_{\gamma }^{\text{tot}}}{k_{\text{B}}}}\text{d}\gamma =\int_{\gamma }{\bar{P}}_{\bar{\gamma }}\text{d}\gamma =1.$$

The fluctuation theorem has been found several times at the end of the last century [7, 8, 28]. Its applicability extends beyond Markovian processes, encompassing deterministic Hamiltonian systems [29] and quantum systems [30]. For example, Jarzynski equality as one of the very first integrated fluctuation theorems [8] reveals a relation between work and free energy change in a nonequilibrium process in which a system is driven from an initial equilibrium distribution **p**_init_ to a final equilibrium distribution **p**_fin_. The work done to the system for different realizations varies due to fluctuations, thus an exact relation between work and free energy change cannot be established. However, an equality exists for the average value based on the fluctuation theorem. For such a process, the total entropy production is Δ*S* ^tot^ = (*W* − Δ*F*)/*T*, where *W* is the work done on the system and Δ*F* = *F*_fin_ − *F*_init_ is the free energy change of the system. Therefore, the detailed fluctuation theorem, Equation (13), leads to Crooks relation for work [28].

(15) $$\frac{P(W)}{\bar{P}(-W)}={\text{e}}^{\beta (W-\Delta F)},$$

where *P*(*W*) is the probability of applying an amount of work *W* during a forward process, and $\bar{P}\left(-W\right)$ is the probability of applying an amount of work −*W* during a time‐reversed process. Similarly, the integrated fluctuation theorem, Equation (14), leads to the Jarzynski equality [8],

(16) $$\langle {\text{e}}^{-\beta W}\rangle ={\text{e}}^{-\beta \Delta F}.$$

By applying the Jensen’s inequality, e^−*β*〈*W*〉^ ≤ 〈e^−*βW*^〉, the second law of thermodynamics is recovered as follows:

(17) ΔF≤〈W〉,

which means that the average work done on the system, taken over all realizations of stochastic trajectories, provides an upper bound for the change in free energy. The equal sign is taken in the quasi‐static limit. This inequality can be seen as a statistical‐level manifestation of the second law of thermodynamics.

On the basis of the fluctuation theorem and incorporating the concept of information by feedback control, Sagawa and Ueda introduced a generalized version of Jarzynski equality [31],

(18) $${\langle e^{-\beta \left(W-\Delta F\right)-I}\rangle }_{\gamma }=1,$$

where *I* is the mutual information introduced by feedback control.

### 2.3 Thermodynamic uncertainty relation

**FIGURE 3:**
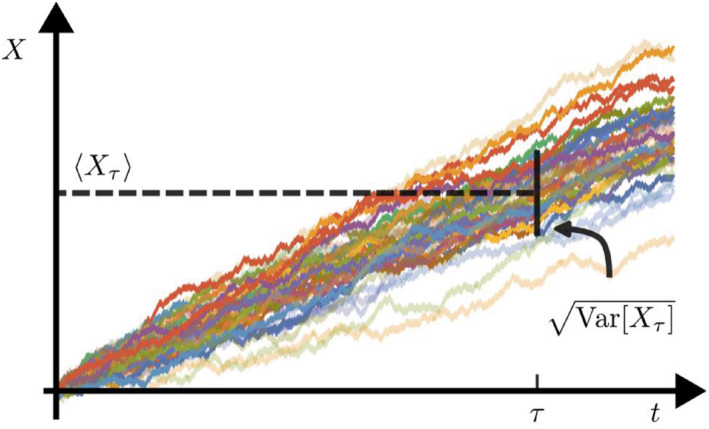
Stochastic trajectories of a biased random walk.

At the mesoscopic scale, physical observables are always subject to fluctuation due to thermal noise. The relation between fluctuations and dissipation is a core focus of stochastic thermodynamics, and the cost of suppressing fluctuation is the central problem of the study of the thermodynamics of biochemical systems. In 2015, Barato and Seifert proposed a universal thermodynamic bound on the fluctuation of stochastic currents [10]. In their work, they studied a biased random walk (a random walk where the probabilities of moving in different directions are not equal) in one dimension to introduce the uncertainty relation of currents. In such a system, the forward and backward transition rates are *k*_+_ and *k*_−_, respectively, which generate stochastic trajectories as shown in Figure 3. Starting from the origin, the mean and variance of the position of the random walker at time *τ* are Var[*X*_*τ*_] = 2*Dτ* = (*k*_+_ + *k*_−_)*τ* and 〈*X*_*τ*_〉 = *vτ* = (*k*_+_ − *k*_−_)*τ*, respectively. The biased nature of random walk is associated with a cost of entropy production according to the LDB condition Equation (4), as Δ*S* = *k*_B_ ln(*k*_+_/*k*_−_) per step. The total entropy production after time *τ* is *Σ*_*τ*_ = 〈*X*_*τ*_〉Δ*S*. Combining these expressions one can find a relation between dissipation and precision as follows:

(19) $$\frac{\mathrm{Var}\left[X_{\tau }\right]}{{\langle X_{\tau }\rangle }^{2}}=\frac{\Delta S/\left(2k_{B}\right)}{\tanh\left[\Delta S/\left(2k_{B}\right)\right]}\frac{2k_{B}}{\Sigma_{\tau }}\geq \frac{2k_{B}}{\Sigma_{\tau }}.$$

It can also be formulated in terms of velocity, diffusion coefficient, and EPR as follows:

(20) $$\frac{D}{v^{2}}\geq \frac{k_{B}}{\dot{\Sigma }},$$

where $\dot{\Sigma }=v\Delta S$ is the EPR.

Although TUR was originally obtained from specific models, it was conjectured to hold for more general stochastic currents in stochastic process, and had later been proven using the large deviation theory in Markov jump processes [32], martingale theory in continuous stochastic process [33], Cramér–Rao bound for multi‐dimensional currents [34, 35] and many other approaches [36, 37, 38]. The general form reads as follows:

(21) $$\frac{\mathrm{Var}\left[J_{\tau }\right]}{{\langle J_{\tau }\rangle }^{2}}\geq \frac{2k_{\text{B}}}{\Sigma_{\tau }},$$

which sets a trade‐off relation between the uncertainty of currents and entropy production. This means that achieving higher precision in a current generally requires a greater dissipation. However, it is worth noting that TUR can be broken in underdamped systems, as illustrated with an example of an underdamped clock [39]. Thus, the validity of TUR is established within the overdamped regime, contingent upon a number of additional assumptions [40]. The extension of TUR beyond the overdamped limit needs to incorporate additional treatments [41, 42, 43, 44].

In addition to TUR, which set a bound on the precision of currents, physicists also focused on the accuracy of clocks in noisy environments, which is directly relevant to the energy cost of maintaining the accuracy of biochemical clocks in living systems [45, 46, 47, 48].

### 2.4 Response around nonequilibrium steady states

A prominent feature of biological systems is their response to the external environment, which is crucial for different biological functions such as sensing, information processing, and copying processes [49, 50, 51]. In equilibrium systems, the probability distribution is given by Boltzmann distribution, which does not contain any kinetics of transition rates, and the response function of a physical observable is related to equilibrium fluctuation by FDT [52]. However, when a system is out of equilibrium, the steady‐state distribution depends not only on the energies of states but also on the kinetics of the transitions, and the FDT can be violated [53, 54].

How do nonequilibrium steady states respond to external perturbations, and what are the constraints imposed by thermodynamics? Recently, a series of works have focused on the general characteristics of nonequilibrium responses, revealing relationships between response sensitivity and factors such as nonequilibrium driving forces, system size, and network topology [11, 22, 55].

The basic idea of these works is to consider the response of the Markov chain around the nonequilibrium steady state, which is determined by the transition rate matrix, *W* **p** ^st^ = 0. The perturbations are classified into three types with a general expression for the transition rates as follows:

(22) $$W_{ij}={\text{e}}^{-F_{ij}-B_{ij}+E_{j}},$$

where *F*_*ij*_ = −*F*_*ji*_ is the edge asymmetric parameter, *B*_*ij*_ is the edge symmetric parameter and *E*_*j*_ is the vertex parameter. By expressing the steady‐state probability using spanning trees, as given in Equation (3), and employing non‐trivial graph‐theoretic manipulations, Owen et al. found universal bounds on different types of perturbations [11]. For example, in the case of edge‐symmetric perturbation, the upper bound of sensitivity is dictated by the nonequilibrium driving force

(23) $$\left|\frac{\partial p_{i}^{\text{st}}}{\partial B_{mn}}\right|\leq p_{i}^{\text{st}}\left(1-p_{i}^{\text{st}}\right)\tanh\left(F_{c}^{\text{max}}/4\right),$$

(24) $$\left|\frac{\partial \;\ln\left(p_{i}^{\text{st}}/p_{j}^{\text{st}}\right)}{\partial B_{mn}}\right|\leq \tanh\left(F_{c}^{\text{max}}/4\right)$$

where $F_{c}^{\text{max}}$ is maximized over all cycles containing the edge e_*mn*_. When detailed balance holds, all cycle affinities vanish, rendering the equilibrium system unable to respond to any edge‐symmetry perturbation (i.e., rescaling a pair of forward and backward rates by the same amount leaves the equilibrium distribution unchanged). This distinction is crucial in differentiating between equilibrium and nonequilibrium systems.

These general response relations can be applied to biological sensing, among other applications. They effectively describe the response of signaling systems, a topic we will discuss in more detail in Section 3.2. In later work, Owen and Horowitz further investigated how the size of a kinetic scheme inherently limits response sensitivity, revealing that even nonequilibrium conditions cannot bypass structural constraints on the effectiveness of biochemical sensitivity mechanisms [22]. More recently, Aslyamov and Esposito provided an alternative derivation of the bounds on response based on simple linear algebra and further obtained bounds on nonequilibrium currents responses [56].

### 2.5 Thermodynamic bounds on spatiotemporal symmetry breaking

Stochastic systems deviate from the equilibrium Boltzmann distribution when the detailed balance is broken. Importantly, this deviation serves as a foundation for spatiotemporal symmetry‐breaking in biological systems such as error correction, pattern formation (e.g., in reaction‐diffusion systems), cross‐membrane transport, and the synchronization of biological clocks [45, 57, 58, 59, 60]. The emergence of such symmetry breaking is closely linked to nonequilibrium driving, which leads to the probability distribution capable of varying in a certain range.

Moreover, a critical difference between equilibrium and nonequilibrium systems lies in their response to external perturbations as discussed in Section 2.4, where a feasible range of steady‐state distribution is essential to allow the response to perturbations on kinetic parameters, unlike at thermodynamic equilibrium, where the steady‐state distribution solely depends on the energies of states and remains unresponsive to kinetic perturbations.

To elucidate the nonequilibrium characteristics of the range, we apply a fundamental inequality, *∑*_*i*_ *y*_*i*_/*∑*_*i*_ *x*_*i*_ ≤ max_*i*_(*y*_*i*_/*x*_*i*_), *∀x*_*i*_ > 0, to the spanning tree steady‐state solution in Equation (3), thereby obtaining the bound on the possible ratio of two steady‐state probabilities [61].

(25) $$\frac{p_{i}^{\text{st}}}{p_{j}^{\text{st}}}=\frac{\sum_{T}w\left(T_{i}\right)}{\sum_{T}w\left(T_{j}\right)}\leq \max_{T}\frac{\;w\left(T_{i}\right)}{w\left(T_{j}\right)}={\text{e}}^{\Delta S_{j\to i}^{\text{env,}\;\text{max}}/k_{\text{B}}}$$

where $\Delta S_{j\to i}^{\text{env,}\;\text{max}}$ is the entropy production into the environment maximized over all paths from state *j* to state *i* in the network. Similarly, the lower bound can be found by minimization and together give the feasible range of steady‐state probability ratio,

(26) $${\text{e}}^{\Delta S_{j\to i}^{\text{env,}\;\text{min}}/k_{\text{B}}}\leq \frac{p_{i}^{\text{st}}}{p_{j}^{\text{st}}}\leq {\text{e}}^{\Delta S_{j\to i}^{\text{env,}\;\text{max}}/k_{\text{B}}},$$

This bound can be further generalized to the ratio of two groups of states, and the case with nonlinear catalytic reaction [23]. Applying it to biological systems leads to the thermodynamic constraints on the accuracy of information processing in biochemical networks, the affinity of chaperone proteins, and the contrast of reaction–diffusion patterns [23, 62, 63, 64].

Furthermore, a recent research study [65] has provided a more general form for the temporal symmetry breaking, which is expressed in terms of propagator between two states, that is, the conditional probability from state *i* to state *j* after a lag time *t*, *P*_*i*|*j*_(*t*), and the reversed one,

(27) $${\text{e}}^{\Delta S_{j\to i}^{\text{env,}\;\text{min}}/k_{\text{B}}}\leq \frac{P_{i|j}(t)}{P_{j|i}(t)}\leq {\text{e}}^{\Delta S_{j\to i}^{\text{env,}\;\text{max}}/k_{\text{B}}},$$

which has the same range as Equation (26) but remains more general as it is not limited to steady states and can be reduced to the Equation (26) in the long time limit as $P_{i|j}\left(\infty \right)=p_{i}^{\text{st}}$. The bound on propagators can further lead to a bound on the asymmetry of cross‐correlations [65]. This, along with other recent results on cross‐correlations [66, 67, 68, 69], allows for the inference of nonequilibrium properties from time‐reversal asymmetry measured through cross‐correlation in biochemical systems [70, 71].

### 2.6 Thermodynamics of CRNs

Chemical reaction networks (CRNs) constitute the foundation of biological processes, playing a crucial role in the emergence of complex phenomena. At thermodynamic equilibrium, a CRN is detailed‐balanced, resulting in a unique and well‐described equilibrium state governed by equilibrium thermodynamics. Understanding the dynamics of out‐of‐equilibrium CRNs, which exhibit rich spatiotemporal behaviors such as multi‐stability and chaos, remains challenging due to their nonlinear nature [72, 73]. Recent advances in nonequilibrium and stochastic thermodynamics provide pivotal insights into studying the thermodynamic properties of out‐of‐equilibrium CRNs at both stochastic and deterministic levels [74, 75, 76]. The introduction of LDB conditions enables the definition of mass‐action kinetics in a thermodynamically consistent way, allowing for the quantification of dissipation in out‐of‐equilibrium CRNs [77].

A CRN involves a set of reactions between different chemical species, each occurring in both forward and backward directions. Methods developed for stochastic Markov processes, such as LDB, can also be introduced to characterize the thermodynamic properties of CRNs [78]. A general CRN is defined as a set of reactions.



(28)

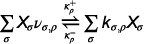

where *ρ* is the index of reaction, $\nu_{\sigma ,\rho }$ and $\kappa_{\sigma ,\rho }$ are the number of chemical species *X*_*σ*_ involved in the forward and backward reactions, respectively. We can define a stoichiometric matrix *S* to describe the reactions, whose element *S*_*σ*,*ρ*_ denotes the change in the number of species *X*_*σ*_ in the reaction *ρ*, defined as follows:

(29) $$S_{\sigma ,\rho }=\kappa_{\sigma ,\rho }-\nu_{\sigma ,\rho }.$$

The reactive semi‐flux of mass‐action kinetics is given as $J_{\rho }^{+}=k_{\rho }^{+}\prod_{\sigma }{\left[X_{\sigma }\right]}^{\nu_{\sigma ,\rho }}$ and $J_{\rho }^{-}=k_{\rho }^{-}\prod_{\sigma }{\left[X_{\sigma }\right]}^{\kappa_{\sigma ,\rho }}$, which combine to give the net flux of reaction *ρ*, $J_{\rho }=J_{\rho }^{+}-J_{\rho }^{-}$. The time evolution of the concentrations is given by the continuity equation as follows:

(30) $$\frac{\text{d}[X]}{\text{d}t}=SJ,$$

where [**X**] represents the vector of concentrations and **J** represents the vector of net fluxes. The thermodynamic structure is introduced through the LDB condition [76].

(31) $$\frac{J_{\rho }^{+}}{J_{\rho }^{-}}={\text{e}}^{-\Delta G_{\rho }/k_{\text{B}}T},$$

where Δ*G*_*ρ*_ = *∑*_*σ*_ *S*_*σ*,*ρ*_(*μ*_*σ*_ − *k*_B_ *T* ln[*X*_*σ*_]) is the Gibbs free energy change of the reaction *ρ*, and *μ*_*σ*_ is the standard chemical potential of species *X*_*σ*_. The entropy production of a chemical reaction system can be written as the sum of net fluxes multiplied by the corresponding free energy changes as follows:

(32) $${\dot{\Sigma }}_{\text{tot}}=R\sum_{\rho }J_{\rho }\;\ln\frac{J_{\rho }^{+}}{J_{\rho }^{-}}.$$

Decomposing dissipation into housekeeping entropy production and excess entropy production for complex‐balanced networks enables the differentiation of the two distinct nonequilibrium sources: the breaking of detailed balance and the process of relaxation [73, 75]. Recent work further refines this decomposition for general nonlinear CRNs [79].

Various mathematical tools have been introduced to study the properties of CRNs. Hessian geometry establishes a Legendre duality between forces and reactive fluxes [80]. Information geometry can reveal the speed limit for the changing rate of the Gibbs free energy [81]. Graph‐theoretic approaches have been employed to identify the thermodynamic bounds on nonequilibrium CRNs, including CRNs’ responses to external perturbations [11, 22, 55, 82], and the accessible chemical space expanded by nonequilibrium driving force [23]. Another avenue involves simplifying complex CRNs through different thermodynamically consistent coarse‐grained approaches, including circuit theory [83] and renormalization group theory [84, 85].

Beyond well‐mixed systems, out‐of‐equilibrium CRN systems with spatial diffusion can exhibit spatiotemporal symmetry breaking such as Turing patterns and chemical waves which are dissipative structures maintained with thermodynamic costs [58, 86]. The development of the phase space geometry approach captures the model‐free properties of mass‐conserving reaction‐diffusion systems [87]. Additionally, recent research has highlighted the role of network topology on Turing pattern formation [88]. Furthermore, chemical reactions in growing systems show the thermodynamic criteria for growth, playing a crucial role in the development of protocells [89].

Despite most studies focusing on an ideal CRN where the dilute approximation excludes molecule interactions, recent research has shifted to the study of nonideal chemical reaction systems, prompted by the discovery of the intracellular condensates [90]. It has been shown that physical interactions can promote spatiotemporal symmetry breaking in reaction–diffusion systems [91, 92], and nonequilibrium chemical reactions can also alter the properties of phase‐separated droplets to achieve effects such as size control, self‐propulsion, and division [93, 94, 95]. Recent efforts have been made to understand different ways to trigger the multi‐stability of non‐ideal chemical reaction systems [96, 97].

## 3 APPLICATIONS TO BIOLOGICAL FUNCTIONS

### 3.1 Fluctuations in molecular machines and intracellular transportation

Stochastic thermodynamics has its origins in the investigation of nanoscale biological machines, specifically motor proteins that transform free energy into mechanical work. These molecular motors—a diverse group of proteins and protein complexes—play crucial roles in myriad biological processes. Based on their working mechanisms, molecular motors can be classified into four categories: linear motors (e.g., kinesin, dynein, and myosin) that move along directed or undirected tracks; rotary motors (e.g., bacterial flagellar motors and ATPases); polymerization motors (e.g., actin and microtubules) that generate force through polymerization; and translocation motors (e.g., DNA packaging motors in bacteriophages) that transport polymers. Brownian ratchet models have been extensively used to study these nanomachines since their popularization by Feynman decades ago [98].

**FIGURE 4:**
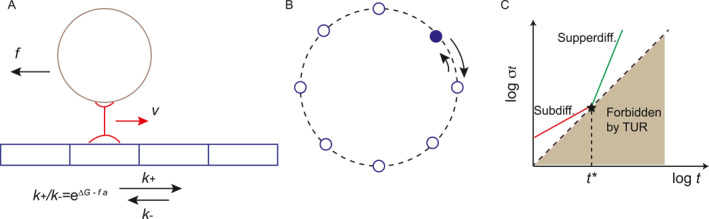
Fluctuations in molecular machines and transportation. (A) A linear motor (red) transporting cargo (brown) along a track. (B) A molecular clock modeled as a biased random walk on a ring. (C) Subdiffusion and superdiffusion with contractions imposed by TUR.

Linear motors, as depicted in Figure 4A, exemplify the class of biological machines that convert chemical free energy, such as the energy released from adenosine triphosphate (ATP) hydrolysis, into mechanical work against opposing forces. These motors exhibit a back‐and‐forth motion on a discrete track due to thermal fluctuations. The motor transitions between steps following a specific rate equation, ensuring LDB.

(33) $$\frac{k_{+}}{k_{-}}={\text{e}}^{-(\Delta G-fa)/k_{\text{B}}T},$$

where Δ*G* is the chemical free energy consumed per step, for example, free energy from ATP hydrolysis, and *fa* is the work done by the motor during a step distance *a*. At steady‐state, the motion exhibits a biased random walk characterized by a mean velocity *v* = *a*(*k*_+_ − *k*_−_) and diffusion coefficient *D* = *a* ^2^(*k*_+_ + *k*_−_)/2. Interestingly, TUR establishes a constraint on the motor’s efficiency *η*, defined as the ratio of work done to the free energy consumed. This constraint, derived in Ref. [100], applies to any linear motor irrespective of its specific mechanochemical details and is expressed by the following inequality:

(34) $$\eta =\frac{fa}{\Delta G}=\frac{fa}{fa+(\Delta G-fa)}\leq \frac{1}{1+vk_{\text{B}}T/Df},$$

where we have used the TUR *D*/*v* ^2^ ≥ *k*_B_ *T*/(Δ*G* − *fa*). This result highlights that the efficiency of a linear motor is inherently limited by both its thermodynamic properties and its kinetic parameters. Notably, the efficiency bound is independent of the specific motor’s details, making it a universal constraint for linear motors. For rotary motors, such as the bacterial flagellar motor, which operates in viscous environments, a different efficiency metric, known as Stokes efficiency, is employed. This concept and detailed mechanisms of rotary motor efficiency are discussed in Ref. [101].

Similar to linear and rotary motors, the behavior of molecular clocks, such as the Kai system in cyanobacteria, can be described as a biased random walk on a ring (Figure 4B). In this case, the position on the track represents the phase of the oscillator. In Ref. [45], the authors demonstrated that, for a broad range of biochemical oscillators, the phase diffusion (analogous to the diffusion coefficient in motor models) exhibits an inverse relationship with the free energy consumed per cycle:

(35) $$D∼\frac{W_{0}}{\Delta W-W_{c}},$$

where *D* is the phase diffusion coefficient, Δ*W* represents the dissipation per period, *W*_0_ and *W*_*c*_ are constants. Notably, this relationship aligns with the TUR, which states that $D/v^{2}\geq k_{\text{B}}T/{\dot{W}}_{ss}$, where *v* is the mean velocity of the random walk and ${\dot{W}}_{ss}$ denotes the steady‐state free energy dissipation rate [102]. This connection between the TUR and biological clocks has been further explored in subsequent studies [46, 48]. Consequently, the TUR imposes a fundamental limit on the coherence, or precision, of molecular clocks, directly linking their performance to their free energy consumption.

In contrast to the linear relationship between displacement variance and time observed in normal diffusion processes $\left(\sigma_{x}^{2}∼t\right)$, cellular environments such as actin networks often exhibit anomalous diffusion, where the variance grows with time according to a power law:

(36) $$\sigma_{x}^{2}\approx K_{a}t^{\alpha }.$$

Here, *K*_*a*_ is the generalized diffusion coefficient, and the exponent *α* determines the anomalous nature of the process (*α* > 1 for superdiffusion, *α* < 1 for subdiffusion).

If this anomalous diffusion is driven by free energy consumption and reaches a steady state, the TUR imposes a fundamental restriction, establishing a linear lower bound for the variance:

$$\sigma_{x}^{2}\geq 2k_{\text{B}}Tv^{2}t/{\dot{W}}_{ss}=Ct,$$

where *v* denotes the mean velocity, ${\dot{W}}_{ss}$ represents the steady‐state free energy dissipation rate, and *C* is a constant. Compare Equation (36) with the TUR bound, a critical time scale t* emerges [99]:

(37) $$t^{*}\approx {\left(\frac{K_{a}}{C}\right)}^{1/\left(1-\alpha \right)}.$$

This critical time scale governs the occurrence of different anomalous regimes. As depicted in Figure 4C, the TUR bound restricts the system to the region above the line, prohibiting subdiffusion for times exceeding t* and superdiffusion for times shorter than t*. Consequently, the TUR establishes a fundamental limit on the time scale for which anomalous kinetics can manifest in finite systems.

Biological systems extensively utilize transport processes fueled by chemical energy, a defining feature of life. While stochastic thermodynamics establishes lower bounds for the efficiency of many biological processes, understanding how real systems approach these theoretical limits remains a major challenge in its application. This gap highlights the need for bridging the knowledge gap between theoretical constraints and the intricate working mechanism of biological systems. Recent advancements in structural biology, particularly the Cryo‐EM technique, have revolutionized our understanding of biological nanomachines at unprecedented molecular detail. For instance, detailed structural analyses of the flagellar motor [103, 104, 105] have shed light on its torque generation and rotational switching mechanisms. Notably, these novel insights have paved the way for the development of new models [106] that provide more realistic constraints on the performance of biological nanomachines.

### 3.2 Proofreading and error correction

**FIGURE 5:**
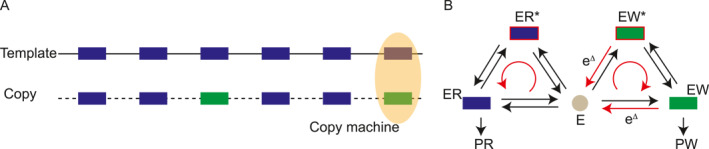
Schemes of proofreading. (A) A template‐replication mechanism depicting correct (blue) and incorrect (green) matches during the replication process. (B) A one‐step kinetic proofreading scheme illustrating the production of correct products (PR) and incorrect products (PW). Reactions labeled with a discrimination factor e^Δ^ highlight the selectivity process.

Maintaining high fidelity during information transfer processes is critical for proper biological function. Examples of such processes include DNA replication, which safeguards genetic stability by precisely copying genetic information; the central dogma of molecular biology, encompassing transcription and translation; and receptor‐ligand binding events, such as those involved in the immune response, where specific recognition is crucial. Thermal fluctuations, however, can introduce errors in these processes leading to “wrong matches” (e.g., mismatched base pairs in DNA replication). As depicted in Figure 5A, in a typical copy process, the error rate *η* can be quantified as the ratio of the number of wrong matches *N*_*w*_ to the number of right matches *N*_*r*_

(38) $$\eta =\frac{N_{w}}{N_{r}}.$$

Equilibrium thermodynamics dictates that *N*_*r*_ and *N*_*w*_ can be given by the Boltzmann distribution, where *N*_*r*, *w*_ ∼ exp(−*E*_*r*,*w*_/*k*_b_ *T*) and *E*_*r*,*w*_ represent the free energies of the right and wrong matches, respectively. Consequently, the equilibrium error rate becomes,

$$\eta_{eq}∼{\text{e}}^{-\left(E_{w}-E_{r}\right)/k_{\text{B}}T}.$$

Based on this framework, the free energy difference Δ = (*E*_*w*_ − *E*_*r*_)/*k*_B_ *T* between wrong and right matches would theoretically yield error rates of 10^−4^ for DNA replication and 10^−2^ for protein synthesis, significantly higher than the observed values in biological systems [107, 108].

Kinetic proofreading, introduced independently by John Hopfield [12] and Jacques Ninio [109] around four decades ago, offers a compelling explanation for the high fidelity observed in biological processes, such as replication, transcription, and translation. This mechanism hinges on the incorporation of an additional, irreversible step within the process. This step amplifies the free energy difference between correct and incorrect matches at the expense of free energy consumption.

As depicted in Figure 5B, kinetic proofreading operates in the regime where nonequilibrium processes dominate. Two reaction pathways lead to the final products (*P*_*R*_ and *P*_*W*_). Crucially, most intermediate complexes (ES) return to the initial state (E + S) instead of proceeding to the final product. This “kinetic filtering” step, fueled by excess free energy, creates a loop that discriminates between right and wrong matches through an additional proofreading process.

Consequently, the error rate *η*, originally defined in Equation (38), can be significantly reduced due to this additional step, reaching a theoretical limit of *η* = e^−2Δ^. Further refinements incorporating multiple, cascading proofreading steps, as explored in Refs. [63, 110] (see Ref. [111] for a detailed review) and generalized in Ref. [22], suggest an even lower error limit

$$\eta ∼{\text{e}}^{-(n+1)\Delta },$$

where *n* represents the number of proofreading steps.

Importantly, studies by Refs. [57, 63] have established a thermodynamic bound on the achievable error rate:

(39) $$\eta \geq \eta_{eq}\;\exp\left(-\frac{\Delta W_{p}+\Delta F}{T}\right).$$

This inequality holds true regardless of the specific proofreading mechanism employed. Here Δ*W*_*p*_ denotes the work dissipated during error correction, and $\Delta F=-k_{\text{B}}T\;\log\left({\text{e}}^{-E_{r}/k_{\text{B}}T}+{\text{e}}^{-E_{w}/k_{\text{B}}T}\right)$ represents the average free energy per added monomer at equilibrium.

However, the cyclic proofreading mechanism suffers from a critical limitation: as the system approaches the error limit, the time required to produce a correct product diverges due to the predominance of the cyclical pathway. Over the past decade, researchers have identified additional regimes within the error correction machinery.

Murugan et al. [110, 112] discovered a kinetic regime that exponentially speeds up the process at the expense of a less optimal error rate. This finding underscores the existence of an inherent trade‐off between accuracy, speed, and energy dissipation within kinetic proofreading networks.

Furthermore, studies by Sartori et al. [49, 57] and Rao et al. [113] revealed that the effectiveness of error correction hinges on the network’s structural topology. Depending on the specific network structure and kinetics, the copy machine can operate in one of three regimes: error correction, error amplification, or a Maxwell demon (extracting work from error correction). Crucially, all three regimes adhere to the following first‐law‐like relation:

(40) $$T\Delta S_{\mathrm{tot}}=\Delta W-\Delta F+D\left(\eta \|\eta_{eq}\right)\geq 0.$$

Here Δ*S*_tot_ represents the total entropy production, Δ*W* denotes the total work performed, and *D*(*η*‖*η*_*eq*_) = *η* log(*η*/*η*_*eq*_) + (1 − *η*) log[(1 − *η*)/(1 − *η*_*eq*_)] is the Kullback–Leibler divergence, quantifying the difference between the equilibrium and nonequilibrium error distributions. This unifying result allows for the analysis of error correction processes within any complex reaction network.

Applying Equation (39) to estimate the error rate of a copy process consuming one Guanosine triphosphate (GTP) molecule, such as DNA replication, yields a value significantly lower than observed in experiments [57]. This observation suggests either (a) the thermodynamic bound is too lenient for DNA replication or (b) not all the free energy released from GTP hydrolysis is utilized for error correction. Several studies [22, 112] have further demonstrated that the effectiveness of proofreading depends heavily on the underlying reaction network’s structure (topology). Unraveling how network topology shapes the capabilities of kinetic proofreading and identifying design principles for achieving high fidelity in biological systems remain open questions.

### 3.3 Sensitivity, adaptation and accuracy in biological sensing

Living cells exhibit remarkable sensitivity, responding to incredibly subtle environmental perturbations. For instance, chemotactic bacteria can detect chemical attractants at concentrations where only a few molecules are present [114]. Similarly, eukaryotic cells can sense differences as small as 10 molecules between their front and rear [115]. Within signal transduction cascades, minor changes in input signals can trigger ultrasensitive activation of downstream outputs [116, 117]. Furthermore, measurements in the gene regulatory networks driving early development in *Drosophila* embryos indicate that positional information is encoded with near‐optimal efficiency, approaching the theoretical limit imposed by the number of available regulatory proteins [118, 119].

**FIGURE 6:**
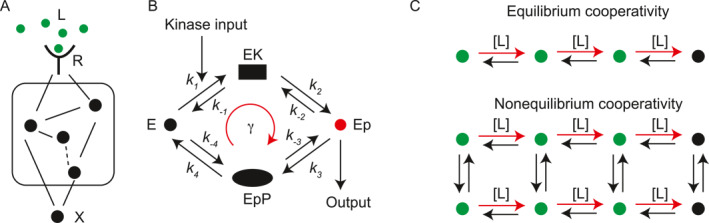
Sensitivity in nonequilibrium biological sensing. (A) Schematic representation of a general sensing network. (B) The phosphorylation‐dephosphorylation cycle responding to kinase concentration. (C) Top: biochemical reaction network representing equilibrium cooperative binding. Green dots depict the support states influenced by ligand concentration. Bottom: nonequilibrium cooperative binding. Detailed balance is broken in the vertical transitions.

As illustrated in Figure 6A, the sensing process can be modeled as a series of biochemical reactions involving the ligand, its binding receptor, a downstream processing network controlled by the receptor, and a readout molecule that reflects the ligand signal. The input signal is represented by the ligand concentration [*L*], while the output is the readout molecule concentration [*X*]. The relationship between input and output is captured by a function [*X*] = *f*([*L*]).

Three key characteristics define a sensory system: sensitivity, accuracy, and detection range. Sensitivity measures the responsiveness of the system to changes in input, quantified as follows:

(41) $$S=\frac{\text{d}\;\log\;f(c)}{\text{d}\;\log\;c}.$$

where *c* represents the ligand concentration. A classic example, the sigmoidal Hill function

(42) $$f\left(c\right)=\frac{c^{n}}{c^{n}+K^{n}}$$

yields a sensitivity of *S* = *n*[1 − *f*(*c*)]. To explain the remarkable sensitivity observed in biological systems, various mechanisms have been proposed, which can be broadly categorized into equilibrium and nonequilibrium strategies.

Several equilibrium mechanisms have been proposed to explain biological sensitivity, including the Monod–Wyman–Changeux (MWC) allosteric model [120] and Ising‐type models [121] (see Ref. [122] for a comprehensive review). These mechanisms rely on the cooperativity between receptors. The concept of allosteric cooperativity was initially introduced by Hill to explain the sigmoidal oxygen‐binding curve of hemoglobin [123]. In the MWC model, a group of *n* tightly coupled receptors functions as a single unit—they are either all activated or all inactive. This collective behavior amplifies the free energy required for receptor cluster activation by a factor of *n*, potentially leading to a response curve like that described in Equation (42). The MWC model can be viewed as a specific Ising‐type model with an infinitely strong coupling within the receptor subcluster.

The MWC model can be extended to nonequilibrium regimes to achieve even higher sensitivity than its equilibrium counterpart [22, 50]. This mechanism has been used to explain the high sensitivity (effective Hill coefficient ≈ 21) observed in the response of the *Escherichia coli* flagellar motor rotation direction (clockwise or counterclockwise) to intracellular CheY‐P concentration [124, 125]. However, many biological systems, such as the mitogen‐activated protein kinase pathway [126] and G‐protein coupled receptors, exhibit ultrasensitivity despite lacking evidence of receptor cooperativity.

In these cases, the biochemical reactions can be modeled as a phosphorylation–dephosphorylation (PdP) cycle, as illustrated in Figure 6B. This cycle comprises two enzymatic reactions catalyzed by a kinase and a phosphatase, with rates given by *k*_±1,±2,±3,±4_. Notably, the dephosphorylation reaction is not the reverse of the phosphorylation reaction; instead, both reactions proceed with their own reverse reactions, as depicted in the figure. Each complete PdP cycle dissipates free energy, quantified by the following equation:

(43) $$\Delta \mu =k_{\text{B}}T\;\ln\frac{k_{1}k_{2}k_{3}k_{4}}{k_{-1}k_{-2}k_{-3}k_{-4}}=k_{\text{B}}T\;\ln\;\gamma .$$

Goldbeter and Koshland [116] were the first to demonstrate that PdP cycles can exhibit ultrasensitivity when the kinase and phosphatase reactions operate in the saturation regime (zeroth‐order kinetics). This corresponds to conditions where the Michaelis constants (*K*_1_ and *K*_2_) are significantly smaller than the total enzyme concentration (*E*_*T*_). Several studies, including [11, 74, 78, 127, 128], have highlighted the role of free energy dissipation Δ*μ* in enhancing sensitivity. Notably, Owen et al. [11] rigorously proved that the sensitivity of any nonequilibrium‐sensing network is bounded by the maximum affinity within its cycles. Applying this finding to the PdP cycle yields the following inequality:

(44) $$S=\frac{\partial \;\ln\left(\left[Ep\right]/\left[E\right]\right)}{\partial \;\ln\left[K\right]}\leq \tanh\left(\Delta \mu /4k_{B}T\right).$$

Here, [*Ep*] denotes the concentration of the phosphorylated enzyme, [*K*] represents the kinase concentration. This bound holds true for any network where the phosphorylated species (*E*_*p*_) is formed through any number of intermediate complexes with arbitrary reaction rates. The upper bound in Equation (44) is a function of free energy dissipation and reduces to zero when Δ*μ* = 0, which indicates detailed balance must be broken to allow a nonzero response.

At thermodynamic equilibrium, cooperative binding serves as the fundamental mechanism for sensitivity. The number of binding sites inherently imposes an upper limit on the effective Hill coefficient. This notion was extended by Owen et al. [22] to encompass any nonequilibrium kinetic sensing scheme. They established that the size of the support (*m*), a structural property of the sensing network, invariably restricts the effective Hill coefficient according to the following inequality:

(45) S≤m.

This principle is the support bound for kinetic schemes. The support encompasses states whose exit rates are directly influenced by the signal. In the context of cooperative binding at equilibrium, the support size simply corresponds to the number of binding sites. For nonequilibrium cooperative binding, the support size represents the number of states that can be regulated by the ligand, potentially doubling the effective Hill coefficient for the scheme depicted in Figure 6C. This limitation applies not only to chemical sensing but also to gene regulatory networks, as demonstrated in Refs. [129, 130, 131]. Interestingly, it has been proposed that energy expenditure can enable Hill coefficients exceeding *n*, even with only *n* transcriptional factor binding sites. This concept could potentially explain the remarkable sharpness observed in gene regulation during *Drosophila* development [119].

A fundamental trade‐off exists between sensitivity and detection range in sensory systems with a fixed number of receptors (*N*). If the cell relies solely on counting occupied receptors, the maximum number of distinguishable outputs is limited by *N*. Consequently, highly sensitive receptors can amplify small changes in ligand concentration but exhaust their detection range in the process. Adaptation mechanisms address this limitation by shifting the input–output curve based on the background signal, allowing them to maintain both high sensitivity and a broad detection range. However, this adaptation comes at an energy cost. Several studies [132, 133, 134] have established a quantitative relationship between energy expenditure (Δ*W*), adaptation speed (*ω*), and the accuracy of adaptation (quantified by the adaptation error, *ϵ*):

(46) $$\dot{W}\approx c_{0}\omega \times \ln\left(\frac{\epsilon_{0}}{\epsilon }\right).$$

Here, *c*_0_ and *ϵ*_0_ are constants. This equation highlights the inherent trade‐off: achieving faster adaptation or higher accuracy requires a higher energy expenditure. A more detailed review on this topic can be found in Ref. [135].

Due to the inherent stochasticity of the chemical sensing process, cells face an irreducible level of uncertainty when inferring the environmental signal. Given an input–output function *f*(*c*) representing the relationship between ligand concentration (*c*) and the readout molecule concentration (*X*), the fractional error in estimating the ligand concentration can be calculated using the rule of error propagation:

(47) $${\left(\frac{\delta c}{c}\right)}^{2}=\frac{\sigma_{X}^{2}}{c^{2}{\left(\text{d}f/\text{d}c\right)}^{2}}=\frac{\sigma_{X}^{2}}{f^{2}S^{2}}.$$

Here, $\sigma_{X}^{2}$ represents the variance associated with the receptor or the readout, and *S* denotes the system’s sensitivity. Burg and Purcell suggested that cells can reduce sensing errors by increasing the number of measurements [136]. This can be achieved either by employing more receptors or by taking more measurements per receptor (effectively, time averaging). In the latter case, the error scales as *T*/(*R*_*T*_ *τ*_*r*_), where *T* is the total measurement time, *τ*_*r*_ is the measurement time per receptor, and *R*_*T*_ is the total receptor number.

Recent studies have revisited the Burg–Purcell limit [137, 138, 139]. For a comprehensive review, see Ref. [51]. Notably, from a nonequilibrium sensing perspective, since the sensing error is inherently linked to sensitivity (as shown in Equation (47)), any mechanism enhancing sensitivity can potentially improve accuracy beyond the equilibrium Burg–Purcell limit [140, 141]. Interestingly, both energetics (Equation 44) and network structure (Equation 45) impose limitations on sensitivity. Consequently, there exists a lower bound for the error:

(48) $${\left(\frac{\delta c}{c}\right)}^{2}\geq \max\left(\frac{4T}{R_{T}\tau_{r}},\frac{4}{X_{T}},\frac{4k_{\text{B}}T}{\Delta \mu }\right).$$

This relationship was first established in Ref. [142]. The three individual limits cannot be compensated by each other. To achieve optimal accuracy, cells need to strategically allocate their sensing resources, ensuring that all three limits become equal, thereby minimizing resource waste [142].

While concentration sensing serves as a well‐studied example of stochasticity in cellular systems, other vital signals beyond simple concentration levels are crucial for cellular function. For instance, eukaryotic cells employ membrane receptors to measure concentration gradients and guide chemotaxis. A crucial question remains: can nonequilibrium effects enhance the sensitivity of gradient sensing? Similarly, circadian clocks exhibit phase response sensitivity, which has been shown to be bounded by energy dissipation [143]. However, translating this bound within the framework of stochastic thermodynamics is an open question.

On the other hand, achieving extremely high sensitivity seems to necessitate a large timescale separation within the sensory system, as demonstrated by Owen et al. [22] and observed in the kinetic proofreading scheme. This suggests a sensitivity‐speed trade‐off in nonequilibrium sensory systems. Unveiling how living systems achieve a balance between these two crucial aspects continues to be a compelling area of research, holding significant implications for both theoretical and experimental endeavors.

### 3.4 Collective behaviors and nonequilibrium phase transitions

A central theme in the field of active matter is the emergence of collective behavior from interacting energy‐consuming components. These active agents, such as the self‐propelled particles in the Vicsek model [144] and the self‐sustained oscillators in the Kuramoto model [145], operate in a nonequilibrium state and require energy input. Since collective behaviors generate order at the population (ensemble) level, it is intuitive to expect that additional free energy expenditure is necessary to achieve alignment of all active agents. While the specific energetic cost of alignment might vary due to the nonequilibrium nature of the dynamics, recent studies suggest a degree of generality in this phenomenon.

In Herpich et al.’s study [146], a thermodynamically consistent model of driven interacting three‐state units was used to investigate oscillation synchronization. The authors observed a Hopf bifurcation to a limit cycle, indicating synchronization at the mean‐field level. By employing stochastic thermodynamics, they discovered that the disordered state exhibits higher dissipation compared to the ordered state. Similarly, Yu et al. [147] studied the active Ising model and revealed the emergence of an additional energy requirement for spin alignment alongside the energy needed for driving motion. Interestingly, they observed a peak in dissipation at the flocking transition onset, followed by lower dissipation in the ordered flocking state.

Building upon the concept of noise molecular clocks (introduced in subsection 3.1), Zhang et al. [148] investigated another model for synchronizing a group of such clocks. While maintaining coherence within individual clocks requires energy expenditure, it is insufficient for generating collective oscillation. Significant fluctuations in the underlying chemical reactions rapidly lead to individual clock desynchronization, and macroscopic oscillation ceases. To address this challenge, the authors introduced a phase‐exchange mechanism inspired by the Kai system, the cyanobacterial circadian clock [149]. This mechanism, analogous to the monomer shuffling observed in the Kai system, couples individual oscillators. Interestingly, synchronous oscillation emerges only when both the interaction strength and frequency exceed a critical value, unique for nonequilibrium phase transition. Notably, this transition requires an excess free energy expenditure, enabling the global synchronization of the oscillators. Furthermore, this relationship between synchronized oscillation and energy dissipation offers a potential explanation for the observed excess ATP consumption in the Kai system beyond the known energy cost associated with KaiC protein phosphorylation and dephosphorylation [150].

While free energy minimization governs self‐assembly in equilibrium systems, the study of collective behaviors in nonequilibrium systems reveals a fascinating interplay between energy dissipation and the emergence of order. This suggests that energy expenditure plays a critical role in how these systems organize and adapt. This active field, applying nonequilibrium thermodynamics to the study of collective behavior, holds the potential to unveil the fundamental principles governing the organization of life within its inherently dynamic environment.

## 4 OUTLOOK

Stochastic thermodynamics aims to establish general constraints on nonequilibrium stochastic processes, predicting their feasibility within the framework of thermodynamics. The resulting thermodynamic bounds can generally be interpreted from two main perspectives: (a) the achievable limits with given nonequilibrium drivings and (b) the minimum energy expenditure of a system inferred from specific observed quantities. Despite the effectiveness of existing thermodynamic bounds in biochemical systems, the application of stochastic thermodynamics to real biological systems often encounters challenges due to the limited experimental access to comprehensive system details and the precision constraints of measurements. Consequently, the energy consumption inferred from these data may not accurately reflect the system’s true behavior. Thus, a major direction in stochastic thermodynamics research is to establish thermodynamic bounds for partially observable quantities [151, 152, 153, 154, 155, 156], coarse‐grained observables [65, 157, 158, 159], and other novel observables [160, 161, 162, 163, 164]. These research efforts aim to strengthen the link between thermodynamic bounds and measurable real‐world systems, thereby facilitating more reliable predictions for real biological systems.

While stochastic thermodynamics offers valuable insights into biological systems, its limitations become apparent when considering the full spectrum of factors shaping biological function. As current research studies reveal, many elements, such as biological network topology, protein structures and communication channels [165], significantly influence performance. These findings suggest that thermodynamics alone might not provide the sole governing principle for understanding the remarkable complexity of life. “Structure determines function” serves as a cornerstone of biology. Yet, fully integrating this principle into current research presents a challenge. To achieve a quantitative understanding of living systems, integrating diverse biological data and constraints into the framework of stochastic thermodynamics becomes crucial.

## AUTHOR CONTRIBUTIONS

**Yuansheng Cao**: Conceptualization; project administration; writing—original draft; writing—review & editing. **Shiling Liang**: Conceptualization; project administration; writing—original draft; writing—review & editing.

## CONFLICT OF INTEREST STATEMENT

The authors declare no conflicts of interest.

## Data Availability

Data sharing is not applicable to this article as no new data were created or analyzed in this study.

## References

[1] Orth JD , Thiele I , Palsson BØ . What is flux balance analysis? Nat Biotechnol. 2010;28(3):245–248. 20212490 10.1038/nbt.1614 PMC3108565

[2] Niebel B , Leupold S , Heinemann M . An upper limit on Gibbs energy dissipation governs cellular metabolism. Nat Metab. 2019;1(1):125–132. 32694810 10.1038/s42255-018-0006-7

[3] Lebowitz JL , Bergmann PG . Irreversible gibbsian ensembles. Ann Phys. 1957;1(1):1–23.

[4] Prigogine I . Introduction to thermodynamics of irreversible processes. New York: John Wiley & Sons; 1967.

[5] Hill T . Free energy transduction in biology: the steady‐state kinetic and thermodynamic formalism. New York: Academic Press; 1977.

[6] Sekimoto K . Stochastic energetics, volume 799 of lecture notes in physics. Berlin, Heidelberg: Springer Berlin Heidelberg; 2010.

[7] Evans DJ , Cohen EGD , Morriss GP . Probability of second law violations in shearing steady states. Phys Rev Lett. 1993;71(15):2401–2404. 10054671 10.1103/PhysRevLett.71.2401

[8] Jarzynski C . Nonequilibrium equality for free energy differences. Phys Rev Lett. 1997;78(14):2690–2693.

[9] Crooks GE . Nonequilibrium measurements of free energy differences for microscopically reversible Markovian systems. J Stat Phys. 1998;90(5):1481–1487.

[10] Barato AC , Seifert U . Thermodynamic uncertainty relation for biomolecular processes. Phys Rev Lett. 2015;114(15):158101. 25933341 10.1103/PhysRevLett.114.158101

[11] Owen JA , Gingrich TR , Horowitz JM . Universal thermodynamic bounds on nonequilibrium response with biochemical applications. Phys Rev X. 2020;10(1):011066.

[12] Hopfield JJ . Kinetic proofreading: a new mechanism for reducing errors in biosynthetic processes requiring high specificity. Proc Natl Acad Sci USA. 1974;71(10):4135–4139. 4530290 10.1073/pnas.71.10.4135 PMC434344

[13] Astumian RD . Thermodynamics and kinetics of a Brownian motor. Science. 1997;276(5314):917–922. 9139648 10.1126/science.276.5314.917

[14] Goloubinoff P , Sassi AS , Fauvet B , Barducci A , De Los Rios P . Chaperones convert the energy from ATP into the nonequilibrium stabilization of native proteins. Nat Chem Biol. 2018;14(4):388–395. 29507388 10.1038/s41589-018-0013-8

[15] Busiello DM , Liang S , Piazza F , De Los Rios P . Dissipation‐driven selection of states in non‐equilibrium chemical networks. Commun Chem. 2021;4(1):16. 36697543 10.1038/s42004-021-00454-w PMC9814615

[16] Seifert U . Entropy production along a stochastic trajectory and an integral fluctuation theorem. Phys Rev Lett. 2005;95(4):040602. 16090792 10.1103/PhysRevLett.95.040602

[17] Seifert U . Stochastic thermodynamics, fluctuation theorems, and molecular machines. Rep Prog Phys. 2012;75(12):126001. 23168354 10.1088/0034-4885/75/12/126001

[18] Peliti L , Pigolotti S . Stochastic thermodynamics: an introduction. Princeton, NJ: Princeton University Press; 2021.

[19] King EL , Altman C . A schematic method of deriving the rate laws for enzyme‐catalyzed reactions. J Phys Chem. 1956;60(10):1375–1378.

[20] Schnakenberg J . Network theory of microscopic and macroscopic behavior of master equation systems. Rev Mod Phys. 1976;48(4):571–585.

[21] Hill TL . Free energy transduction and biochemical cycle kinetics. New York: Springer; 1989.

[22] Owen JA , Horowitz JM . Size limits the sensitivity of kinetic schemes. Nat Commun. 2023;14(1):1280. 36890153 10.1038/s41467-023-36705-8 PMC9995461

[23] Liang S , De Los Rios P , Busiello DM . Thermodynamic bounds on symmetry breaking in linear and catalytic biochemical systems. Phys Rev Lett. 2024;132(22):228402. 38877915 10.1103/PhysRevLett.132.228402

[24] Nam K‐M , Gunawardena J . The linear framework II: using graph theory to analyse the transient regime of Markov processes. Front Cell Dev Biol. 2023;11:1233808. 38020901 10.3389/fcell.2023.1233808 PMC10656611

[25] Maes C . Local detailed balance. SciPost Phys Lect Notes. 2021:32.

[26] Kolmogoroff A . Zur Theorie der Markoffschen Ketten. Math Ann. 1936;112(1):155–160.

[27] Esposito M , Van den Broeck C . Three detailed fluctuation theorems. Phys Rev Lett. 2010;104(9):090601. 20366974 10.1103/PhysRevLett.104.090601

[28] Crooks GE . Entropy production fluctuation theorem and the nonequilibrium work relation for free energy differences. Phys Rev. 1999;60(3):2721–2726. 10.1103/physreve.60.2721 11970075

[29] Jarzynski C . Hamiltonian derivation of a detailed fluctuation theorem. J Stat Phys. 2000;98(1):77–102.

[30] Åberg J . Fully quantum fluctuation theorems. Phys Rev X. 2018;8(1):011019.

[31] Sagawa T , Ueda M . Generalized Jarzynski equality under nonequilibrium feedback control. Phys Rev Lett. 2010;104(9):090602. 20366975 10.1103/PhysRevLett.104.090602

[32] Gingrich TR , Horowitz JM , Perunov N , England JL . Dissipation bounds all steady‐state current fluctuations. Phys Rev Lett. 2016;116(12):120601. 27058064 10.1103/PhysRevLett.116.120601

[33] Pigolotti S , Neri I , Roldán É , Jülicher F . Generic properties of stochastic entropy production. Phys Rev Lett. 2017;119(14):140604. 29053318 10.1103/PhysRevLett.119.140604

[34] Dechant A . Multidimensional thermodynamic uncertainty relations. J Phys Math Theor. 2018;52(3):035001.

[35] Dechant A , Sasa S‐I . Fluctuation–response inequality out of equilibrium. Proc Natl Acad Sci USA. 2020;117(12):6430–6436. 32152124 10.1073/pnas.1918386117 PMC7104339

[36] Hasegawa Y , Van Vu T . Uncertainty relations in stochastic processes: an information inequality approach. Phys Rev. 2019;99(6):062126. 10.1103/PhysRevE.99.062126 31330674

[37] Dieball C , Godec A . Direct route to thermodynamic uncertainty relations and their saturation. Phys Rev Lett. 2023;130(8):087101. 36898097 10.1103/PhysRevLett.130.087101

[38] Ziyin L , Ueda M . Universal thermodynamic uncertainty relation in nonequilibrium dynamics. Phys Rev Res. 2023;5(1):013039.

[39] Pietzonka P . Classical pendulum clocks break the thermodynamic uncertainty relation. Phys Rev Lett. 2022;128(13):130606. 35426718 10.1103/PhysRevLett.128.130606

[40] Horowitz JM , Gingrich TR . Thermodynamic uncertainty relations constrain non‐equilibrium fluctuations. Nat Phys. 2020;16(1):15–20.

[41] Van Vu T , Hasegawa Y . Uncertainty relations for underdamped Langevin dynamics. Phys Rev. 2019;100(3):032130. 10.1103/PhysRevE.100.032130 31640023

[42] Lee JS , Park J‐M , Park H . Thermodynamic uncertainty relation for underdamped Langevin systems driven by a velocity‐dependent force. Phys Rev. 2019;100(6):062132. 10.1103/PhysRevE.100.062132 31962517

[43] Plati A , Puglisi A , Sarracino A . Thermodynamic bounds for diffusion in nonequilibrium systems with multiple timescales. Phys Rev. 2023;107(4):044132. 10.1103/PhysRevE.107.044132 37198828

[44] Plati A , Puglisi A , Sarracino A . Thermodynamic uncertainty relations in the presence of non‐linear friction and memory. J Phys Math Theor. 2024;57(15):155001.

[45] Cao Y , Wang H , Ouyang Q , Tu Y . The free‐energy cost of accurate biochemical oscillations. Nat Phys. 2015;11(9):772–778. 26566392 10.1038/nphys3412 PMC4638330

[46] Barato AC , Seifert U . Coherence of biochemical oscillations is bounded by driving force and network topology. Phys Rev. 2017;95(6):062409. 10.1103/PhysRevE.95.062409 28709274

[47] Oberreiter L , Seifert U , Barato AC . Universal minimal cost of coherent biochemical oscillations. Phys Rev. 2022;106(1):014106. 10.1103/PhysRevE.106.014106 35974563

[48] Marsland R , Cui W , Horowitz JM . The thermodynamic uncertainty relation in biochemical oscillations. J R Soc Interface. 2019;16(154):20190098. 31039695 10.1098/rsif.2019.0098 PMC6544898

[49] Sartori P , Pigolotti S . Kinetic versus energetic discrimination in biological copying. Phys Rev Lett. 2013;110(18):188101. 23683246 10.1103/PhysRevLett.110.188101

[50] Tu Y . The nonequilibrium mechanism for ultrasensitivity in a biological switch: sensing by Maxwell’s demons. Proc Natl Acad Sci USA. 2008;105(33):11737–11741. 18687900 10.1073/pnas.0804641105 PMC2575293

[51] ten Wolde PR , Becker NB , Ouldridge TE , Mugler A . Fundamental limits to cellular sensing. J Stat Phys. 2016;162(5):1395–1424.

[52] Marconi UMB , Puglisi A , Rondoni L , Vulpiani A . Fluctuation–dissipation: response theory in statistical physics. Phys Rep. 2008;461(4):111–195.

[53] Harada T , Sasa S‐I . Equality connecting energy dissipation with a violation of the fluctuation‐response relation. Phys Rev Lett. 2005;95(13):130602. 16197127 10.1103/PhysRevLett.95.130602

[54] Harada T , Sasa S‐I . Energy dissipation and violation of the fluctuation‐response relation in nonequilibrium Langevin systems. Phys Rev. 2006;73(2):026131. 10.1103/PhysRevE.73.026131 16605422

[55] Fernandes Martins G , Horowitz JM . Topologically constrained fluctuations and thermodynamics regulate nonequilibrium response. Phys Rev. 2023;108(4):044113. 10.1103/PhysRevE.108.044113 37978593

[56] Aslyamov T , Esposito M . Nonequilibrium response for Markov jump processes: exact results and tight bounds. Phys Rev Lett. 2024;132(3):037101. 38307069 10.1103/PhysRevLett.132.037101

[57] Sartori P , Pigolotti S . Thermodynamics of error correction. Phys Rev X. 2015;5(4):041039.

[58] Avanzini F , Falasco G , Esposito M . Thermodynamics of chemical waves. J Chem Phys. 2019;151(23):234103. 31864268 10.1063/1.5126528

[59] Liang S , Busiello DM , De Los Rios P . Emergent thermophoretic behavior in chemical reaction systems. New J Phys. 2022;24(12):123006.

[60] Flatt S , Busiello DM , Zamuner S , De Los Rios P . ABC transporters are billion‐year‐old Maxwell Demons. Commun Phys. 2023;6(1):205. 38665399 10.1038/s42005-023-01320-y PMC11041718

[61] Maes C , Netočný K . Heat bounds and the blowtorch theorem. Ann Henri Poincaré. 2013;14(5):1193–1202.

[62] Çetiner U , Gunawardena J . Reformulating nonequilibrium steady states and generalized Hopfield discrimination. Phys Rev. 2022;106(6):064128. 10.1103/PhysRevE.106.064128 36671125

[63] Qian H . Reducing intrinsic biochemical noise in cells and its thermodynamic limit. J Mol Biol. 2006;362(3):387–392. 16934833 10.1016/j.jmb.2006.07.068

[64] Nguyen B , Hartich D , Seifert U , De Los Rios P . Thermodynamic bounds on the ultra‐ and infra‐affinity of Hsp70 for its substrates. Biophys J. 2017;113(2):362–370. 28746847 10.1016/j.bpj.2017.06.010 PMC5529314

[65] Liang S , Pigolotti S . Thermodynamic bounds on time‐reversal asymmetry. Phys Rev. 2023;108(6):L062101. 10.1103/PhysRevE.108.L062101 38243435

[66] Ohga N , Ito S , Kolchinsky A . Thermodynamic bound on the asymmetry of cross‐correlations. Phys Rev Lett. 2023;131(7):077101. 37656850 10.1103/PhysRevLett.131.077101

[67] Shiraishi N . Entropy production limits all fluctuation oscillations. Phys Rev. 2023;108(4):L042103. 10.1103/PhysRevE.108.L042103 37978716

[68] Van Vu T , Vo VT , Saito K . Dissipation bounds asymmetry of finite‐time cross‐correlations. Phys Rev Res. 2024;6(1):013273.

[69] Gu J . Thermodynamic bounds on the asymmetry of cross‐correlations with dynamical activity and entropy production. Phys Rev. 2024;109(4):L042101. 10.1103/PhysRevE.109.L042101 38755893

[70] Qian H , Elson EL . Fluorescence correlation spectroscopy with high‐order and dual‐color correlation to probe nonequilibrium steady states. Proc Natl Acad Sci USA. 2004;101(9):2828–2833. 14970342 10.1073/pnas.0305962101 PMC365705

[71] Bacanu A , Pelletier JF , Jung Y , Fakhri N . Inferring scale‐dependent non‐equilibrium activity using carbon nanotubes. Nat Nanotechnol. 2023;18(8):905–911. 37157022 10.1038/s41565-023-01395-2

[72] Feinberg M . Foundations of chemical reaction network theory, volume 202 of applied mathematical sciences. Cham: Springer International Publishing; 2019.

[73] Esposito M . Open questions on nonequilibrium thermodynamics of chemical reaction networks. Commun Chem. 2020;3(1):107. 36703333 10.1038/s42004-020-00344-7 PMC9814766

[74] Qian H . Phosphorylation energy hypothesis: open chemical systems and their biological functions. Annu Rev Phys Chem. 2007;58(1):113–142. 17059360 10.1146/annurev.physchem.58.032806.104550

[75] Ge H , Qian H . Nonequilibrium thermodynamic formalism of nonlinear chemical reaction systems with Waage‐Guldberg’s law of mass action. Chem Phys. 2016;472:241–248.

[76] Rao R , Esposito M . Nonequilibrium thermodynamics of chemical reaction networks: wisdom from stochastic thermodynamics. Phys Rev X. 2016;6(4):041064.

[77] Qian H , Beard DA . Thermodynamics of stoichiometric biochemical networks in living systems far from equilibrium. Biophys Chem. 2005;114(2‐3):213–220. 15829355 10.1016/j.bpc.2004.12.001

[78] Qian H . Thermodynamic and kinetic analysis of sensitivity amplification in biological signal transduction. Biophys Chem. 2003;105(2‐3):585–593. 14499920 10.1016/s0301-4622(03)00068-1

[79] Yoshimura K , Kolchinsky A , Dechant A , Ito S . Housekeeping and excess entropy production for general nonlinear dynamics. Phys Rev Res. 2023;5(1):013017.

[80] Kobayashi TJ , Loutchko D , Kamimura A , Sughiyama Y . Geometry of nonequilibrium chemical reaction networks and generalized entropy production decompositions. 2022.

[81] Yoshimura K , Ito S . Information geometric inequalities of chemical thermodynamics. Phys Rev Res. 2021;3(1):013175.

[82] Chun H‐M , Horowitz JM . Trade‐offs between number fluctuations and response in nonequilibrium chemical reaction networks. J Chem Phys. 2023;158(17):174115. 37144710 10.1063/5.0148662

[83] Avanzini F , Freitas N , Esposito M . Circuit theory for chemical reaction networks. Phys Rev X. 2023;13(2):021041.

[84] Yu Q , Zhang D , Tu Y . Inverse power law scaling of energy dissipation rate in nonequilibrium reaction networks. Phys Rev Lett. 2021;126(8):080601. 33709722 10.1103/PhysRevLett.126.080601 PMC8286115

[85] Yu Q , Tu Y . State‐space renormalization group theory of nonequilibrium reaction networks: exact solutions for hypercubic lattices in arbitrary dimensions. Phys Rev E. 2022;105(4):044140. 35590650 10.1103/PhysRevE.105.044140 PMC9223417

[86] Falasco G , Rao R , Esposito M . Information thermodynamics of Turing patterns. Phys Rev Lett. 2018;121(10):108301. 30240244 10.1103/PhysRevLett.121.108301

[87] Brauns F , Halatek J , Frey E . Phase‐space geometry of mass‐conserving reaction‐diffusion dynamics. Phys Rev X. 2020;10(4):041036.

[88] Diego X , Marcon L , Müller P , Sharpe J . Key features of Turing systems are determined purely by network topology. Phys Rev X. 2018;8(2):021071.

[89] Sughiyama Y , Kamimura A , Loutchko D , Kobayashi TJ . Chemical thermodynamics for growing systems. Phys Rev Res. 2022;4(3):033191.

[90] Brangwynne CP , Eckmann CR , Courson DS , Rybarska A , Hoege C , Gharakhani J , et al. Germline P granules are liquid droplets that localize by controlled dissolution/condensation. Science. 2009;324(5935):1729–1732. 19460965 10.1126/science.1172046

[91] Luo C , Zwicker D . Influence of physical interactions on spatiotemporal patterns. Phys Rev. 2023;108(3):034206. 10.1103/PhysRevE.108.034206 37849174

[92] Menou L , Luo C , Zwicker D . Physical interactions in non‐ideal fluids promote Turing patterns. J R Soc Interface. 2023;20(204):20230244. 37434500 10.1098/rsif.2023.0244 PMC10336379

[93] Zwicker D , Hyman AA , Jülicher F . Suppression of Ostwald ripening in active emulsions. Phys Rev. 2015;92(1):012317. 10.1103/PhysRevE.92.012317 26274171

[94] Zwicker D , Seyboldt R , Weber CA , Hyman AA , Jülicher F . Growth and division of active droplets provides a model for protocells. Nat Phys. 2017;13(4):408–413.

[95] Demarchi L , Goychuk A , Maryshev I , Frey E . Enzyme‐enriched condensates show self‐propulsion, positioning, and coexistence. Phys Rev Lett. 2023;130(12):128401. 37027840 10.1103/PhysRevLett.130.128401

[96] Aslyamov T , Avanzini F , Fodor É , Esposito M . Nonideal reaction‐diffusion systems: multiple routes to instability. Phys Rev Lett. 2023;131(13):138301. 37832019 10.1103/PhysRevLett.131.138301

[97] Miangolarra AM , Castellana M . On non‐ideal chemical‐reaction networks and phase separation. J Stat Phys. 2022;190(1):23.

[98] Feynman RP , Leighton RB , Sands M . The Feynman lectures on physics; vol. I. Am J Phys. 1965;33(9):750–752.

[99] Hartich D , Godec A . Thermodynamic uncertainty relation bounds the extent of anomalous diffusion. Phys Rev Lett. 2021;127(8):080601. 34477441 10.1103/PhysRevLett.127.080601

[100] Pietzonka P , Barato AC , Seifert U . Universal bound on the efficiency of molecular motors. J Stat Mech Theor Exp. 2016;2016(12):124004.

[101] Tu Y , Cao Y . Design principles and optimal performance for molecular motors under realistic constraints. Phys Rev. 2018;97(2):022403. 10.1103/PhysRevE.97.022403 PMC6023414 29548155

[102] Barato AC , Seifert U . Cost and precision of Brownian clocks. Phys Rev X. 2016;6(4):041053.

[103] Deme JC , Johnson S , Vickery O , Aron A , Monkhouse H , Griffiths T , et al. Structures of the stator complex that drives rotation of the bacterial flagellum. Nat Microbiol. 2020;5(12):1553–1564. 32929189 10.1038/s41564-020-0788-8 PMC7610383

[104] Santiveri M , Roa‐Eguiara A , Kühne C , Wadhwa N , Hu H , Berg HC , et al. Structure and function of stator units of the bacterial flagellar motor. Cell. 2020;183(1):244–257. 32931735 10.1016/j.cell.2020.08.016

[105] Chang Y , Zhang K , Carroll BL , Zhao X , Charon NW , Norris SJ , et al. Molecular mechanism for rotational switching of the bacterial flagellar motor. Nat Struct Mol Biol. 2020;27(11):1041–1047. 32895555 10.1038/s41594-020-0497-2 PMC8129871

[106] Cao Y , Li T , Tu Y . Modeling bacterial flagellar motor with new structure information: rotational dynamics of two interacting protein nano‐rings. Front Microbiol. 2022;13:866141. 35694287 10.3389/fmicb.2022.866141 PMC9175137

[107] Johnson KA . Conformational coupling in DNA polymerase fidelity. Annu Rev Biochem. 1993;62(1):685–713. 7688945 10.1146/annurev.bi.62.070193.003345

[108] Hani SZ , Green R . Fidelity at the molecular level: lessons from protein synthesis. Cell. 2009;136(4):746–762. 19239893 10.1016/j.cell.2009.01.036 PMC3691815

[109] Ninio J . Kinetic amplification of enzyme discrimination. Biochimie. 1975;57(5):587–595. 1182215 10.1016/s0300-9084(75)80139-8

[110] Murugan A , Huse DA , Leibler S . Speed, dissipation, and error in kinetic proofreading. Proc Natl Acad Sci USA. 2012;109(30):12034–12039. 22786930 10.1073/pnas.1119911109 PMC3409783

[111] Ge H , Qian M , Qian H . Stochastic theory of nonequilibrium steady states. Part II: applications in chemical biophysics. Phys Rep. 2012;510(3):87–118.

[112] Murugan A , Huse DA , Leibler S . Discriminatory proofreading regimes in nonequilibrium systems. Phys Rev X. 2014;4(2):021016.

[113] Rao R , Peliti L . Thermodynamics of accuracy in kinetic proofreading: dissipation and efficiency trade‐offs. J Stat Mech Theor Exp. 2015;2015(6):P060011.

[114] Sourjik V , Berg HC . Receptor sensitivity in bacterial chemotaxis. Proc Natl Acad Sci USA. 2002;99(1):123–127. 11742065 10.1073/pnas.011589998 PMC117525

[115] Ueda M , Shibata T . Stochastic signal processing and transduction in chemotactic response of eukaryotic cells. Biophys J. 2007;93(1):11–20. 17416630 10.1529/biophysj.106.100263 PMC1914446

[116] Goldbeter A , Koshland DE Jr . An amplified sensitivity arising from covalent modification in biological systems. Proc Natl Acad Sci USA. 1981;78(11):6840–6844. 6947258 10.1073/pnas.78.11.6840 PMC349147

[117] Ferrell JE , Ha SH . Ultrasensitivity part III: cascades, bistable switches, and oscillators. Trends Biochem Sci. 2014;39(12):612–618. 25456048 10.1016/j.tibs.2014.10.002 PMC4254632

[118] Dubuis JO , Tkačik G , Wieschaus EF , Gregor T , Bialek W . Positional information, in bits. Proc Natl Acad Sci USA. 2013;110(41):16301–16308. 24089448 10.1073/pnas.1315642110 PMC3799327

[119] Gregor T , Tank DW , Wieschaus EF , Bialek W . Probing the limits to positional information. Cell. 2007;130(1):153–164. 17632062 10.1016/j.cell.2007.05.025 PMC2253670

[120] Bacon F , Wyman J , Changeux JP . On the nature of allosteric transitions: a plausible model. J Mol Biol. 1965;12(1):88–118. 14343300 10.1016/s0022-2836(65)80285-6

[121] Duke TAJ , Le Novere N , Bray D . Conformational spread in a ring of proteins: a stochastic approach to allostery. J Mol Biol. 2001;308(3):541–553. 11327786 10.1006/jmbi.2001.4610

[122] Tu Y . Quantitative modeling of bacterial chemotaxis: signal amplification and accurate adaptation. Annu Rev Biophys. 2013;42(1):337–359. 23451887 10.1146/annurev-biophys-083012-130358 PMC3737589

[123] Hill AV . The possible effects of the aggregation of the molecules of hemoglobin on its dissociation curves. J Physiol. 1910;40:iv–vii.

[124] Cluzel P , Surette M , Leibler S . An ultrasensitive bacterial motor revealed by monitoring signaling proteins in single cells. Science. 2000;287(5458):1652–1655. 10698740 10.1126/science.287.5458.1652

[125] Yuan J , Berg HC . Ultrasensitivity of an adaptive bacterial motor. J Mol Biol. 2013;425(10):1760–1764. 23454041 10.1016/j.jmb.2013.02.016 PMC3830563

[126] Huang C‐Y , Ferrell JE Jr . Ultrasensitivity in the mitogen‐activated protein kinase cascade. Proc Natl Acad Sci USA. 1996;93(19):10078–10083. 8816754 10.1073/pnas.93.19.10078 PMC38339

[127] Hartich D , Barato AC , Seifert U . Nonequilibrium sensing and its analogy to kinetic proofreading. New J Phys. 2015;17(5):055026.

[128] Skoge M , Naqvi S , Meir Y , Wingreen NS . Chemical sensing by nonequilibrium cooperative receptors. Phys Rev Lett. 2013;110(24):248102. 25165963 10.1103/PhysRevLett.110.248102 PMC4114058

[129] Estrada J , Wong F , DePace A , Gunawardena J . Information integration and energy expenditure in gene regulation. Cell. 2016;166(1):234–244. 27368104 10.1016/j.cell.2016.06.012 PMC4930556

[130] Wong F , Gunawardena J . Gene regulation in and out of equilibrium. Annu Rev Biophys. 2020;49(1):199–226. 32375018 10.1146/annurev-biophys-121219-081542

[131] Mahdavi S , Salmon GL , Daghlian P , Garcia HG , Phillips R . Flexibility and sensitivity in gene regulation out of equilibrium. 2023. Preprint at bioRxiv: 2023.04.11.536490. 10.1073/pnas.2411395121 PMC11573582 39499638

[132] Lan G , Sartori P , Neumann S , Sourjik V , Tu Y . The energy–speed–accuracy trade‐off in sensory adaptation. Nat Phys. 2012;8(5):422–428. 22737175 10.1038/nphys2276 PMC3378065

[133] Lan G , Tu Y . The cost of sensitive response and accurate adaptation in networks with an incoherent type‐1 feed‐forward loop. J R Soc Interface. 2013;10(87):20130489. 23883955 10.1098/rsif.2013.0489 PMC3758009

[134] Sartori P , Granger L , Lee CF , Horowitz JM . Thermodynamic costs of information processing in sensory adaptation. PLoS Comput Biol. 2014;10(12):e1003974. 25503948 10.1371/journal.pcbi.1003974 PMC4263364

[135] Tu Y , Rappel W‐J . Adaptation in living systems. Annu Rev Condens Matter Phys. 2018;9(1):183–205. 30057689 10.1146/annurev-conmatphys-033117-054046 PMC6060625

[136] Berg HC , Purcell EM . Physics of chemoreception. Biophys J. 1977;20(2):193–219. 911982 10.1016/S0006-3495(77)85544-6 PMC1473391

[137] Bialek W , Setayeshgar S . Physical limits to biochemical signaling. Proc Natl Acad Sci USA. 2005;102(29):10040–10045. 16006514 10.1073/pnas.0504321102 PMC1177398

[138] Endres RG , Wingreen NS . Maximum likelihood and the single receptor. Phys Rev Lett. 2009;103(15):158101. 19905667 10.1103/PhysRevLett.103.158101

[139] Govern CC , ten Wolde PR . Fundamental limits on sensing chemical concentrations with linear biochemical networks. Phys Rev Lett. 2012;109(21):218103. 23215617 10.1103/PhysRevLett.109.218103

[140] Lang AH , Fisher CK , Mora T , Mehta P . Thermodynamics of statistical inference by cells. Phys Rev Lett. 2014;113(14):148103. 25325665 10.1103/PhysRevLett.113.148103

[141] Govern CC , ten Wolde PR . Energy dissipation and noise correlations in biochemical sensing. Phys Rev Lett. 2014;113(25):258102. 25554909 10.1103/PhysRevLett.113.258102

[142] Govern CC , Ten Wolde PR . Optimal resource allocation in cellular sensing systems. Proc Natl Acad Sci USA. 2014;111(49):17486–17491. 25422473 10.1073/pnas.1411524111 PMC4267345

[143] Fei C , Cao Y , Ouyang Q , Tu Y . Design principles for enhancing phase sensitivity and suppressing phase fluctuations simultaneously in biochemical oscillatory systems. Nat Commun. 2018;9(1):1434. 29651016 10.1038/s41467-018-03826-4 PMC5897384

[144] Vicsek T , Czirók A , Ben‐Jacob E , Cohen I , Shochet O . Novel type of phase transition in a system of self‐driven particles. Phys Rev Lett. 1995;75(6):1226–1229. 10060237 10.1103/PhysRevLett.75.1226

[145] Kuramoto Y . Chemical oscillations, waves and chemical turbulence. Heidelberg: Springer; 1984.

[146] Herpich T , Thingna J , Esposito M . Collective power: minimal model for thermodynamics of nonequilibrium phase transitions. Phys Rev X. 2018;8(3):031056.

[147] Yu Q , Tu Y . Energy cost for flocking of active spins: the cusped dissipation maximum at the flocking transition. Phys Rev Lett. 2022;129(27):278001. 36638284 10.1103/PhysRevLett.129.278001 PMC10317207

[148] Zhang D , Cao Y , Ouyang Q , Tu Y . The energy cost and optimal design for synchronization of coupled molecular oscillators. Nat Phys. 2020;16(1):95–100. 32670386 10.1038/s41567-019-0701-7 PMC7363412

[149] Kageyama H , Nishiwaki T , Nakajima M , Iwasaki H , Oyama T , Kondo T . Cyanobacterial circadian pacemaker: Kai protein complex dynamics in the KaiC phosphorylation cycle in vitro. Mol Cell. 2006;23(2):161–171. 16857583 10.1016/j.molcel.2006.05.039

[150] Terauchi K , Kitayama Y , Nishiwaki T , Miwa K , Murayama Y , Oyama T , et al. ATPase activity of KaiC determines the basic timing for circadian clock of cyanobacteria. Proc Natl Acad Sci USA. 2007;104(41):16377–16381. 17901204 10.1073/pnas.0706292104 PMC2042214

[151] Shiraishi N , Sagawa T . Fluctuation theorem for partially masked nonequilibrium dynamics. Phys Rev. 2015;91(1):012130. 10.1103/PhysRevE.91.012130 25679593

[152] Martínez IA , Bisker G , Horowitz JM , Parrondo JMR . Inferring broken detailed balance in the absence of observable currents. Nat Commun. 2019;10(1):3542. 31387988 10.1038/s41467-019-11051-w PMC6684597

[153] Lucente D , Baldassarri A , Puglisi A , Vulpiani A , Viale M . Inference of time irreversibility from incomplete information: linear systems and its pitfalls. Phys Rev Res. 2022;4(4):043103.

[154] Harunari PE , Dutta A , Polettini M , Roldán É . What to learn from a few visible transitions’ statistics? Phys Rev X. 2022;12(4):041026.

[155] Van Der Meer J , Degünther J , Seifert U . Time‐resolved statistics of snippets as general framework for model‐free entropy estimators. Phys Rev Lett. 2023;130(25):257101. 37418719 10.1103/PhysRevLett.130.257101

[156] Pietzonka P , Coghi F . Thermodynamic cost for precision of general counting observables. 2023. Preprint at arXiv:2305.15392. 10.1103/PhysRevE.109.064128 39020906

[157] Van Der Meer J , Ertel B , Seifert U . Thermodynamic inference in partially accessible Markov networks: a unifying perspective from transition‐based waiting time distributions. Phys Rev X. 2022;12(3):031025.

[158] Nitzan E , Ghosal A , Bisker G . Universal bounds on entropy production inferred from observed statistics. Phys Rev Res. 2023;5(4):043251.

[159] Blom K , Song K , Vouga E , Godec A , Makarov DE . Milestoning estimators of dissipation in systems observed at a coarse resolution. Proc Natl Acad Sci USA. 2024;121(17):e2318333121. 38625949 10.1073/pnas.2318333121 PMC11047069

[160] Dominic J , Dunkel J . Skinner and Jörn Dunkel. Estimating entropy production from waiting time distributions. Phys Rev Lett. 2021;127(19):198101. 34797138 10.1103/PhysRevLett.127.198101

[161] Kolchinsky A , Ohga N , Ito S . Thermodynamic bound on spectral perturbations, with applications to oscillations and relaxation dynamics. Phys Rev Res. 2024;6(1):013082.

[162] Dechant A , Garnier‐Brun J , Sasa S‐I . Thermodynamic bounds on correlation times. Phys Rev Lett. 2023;131(16):167101. 37925711 10.1103/PhysRevLett.131.167101

[163] Tjalma AJ , Galstyan V , Goedhart J , Slim L , Becker NB , ten Wolde PR . Trade‐offs between cost and information in cellular prediction. Proc Natl Acad Sci USA. 2023;120(41):e2303078120. 37792515 10.1073/pnas.2303078120 PMC10576116

[164] Degünther J , Van Der Meer J , Seifert U . Fluctuating entropy production on the coarse‐grained level: inference and localization of irreversibility. Phys Rev Res. 2024;6(2):023175.

[165] Bryant SJ , Machta BB . Physical constraints in intracellular signaling: the cost of sending a bit. Phys Rev Lett. 2023;131(6):068401. 37625074 10.1103/PhysRevLett.131.068401 PMC11146629

